# Herpes simplex virus infection in pregnancy and in neonate: status of art of epidemiology, diagnosis, therapy and prevention

**DOI:** 10.1186/1743-422X-6-40

**Published:** 2009-04-06

**Authors:** Elena Anzivino, Daniela Fioriti, Monica Mischitelli, Anna Bellizzi, Valentina Barucca, Fernanda Chiarini, Valeria Pietropaolo

**Affiliations:** 1 Department of Public Health Sciences, Sapienza University, Rome, Italy; 2 Department of Urology, Sapienza University, Rome, Italy

## Abstract

Herpes simplex virus (HSV) infection is one of the most common viral sexually transmitted diseases worldwide. The first time infection of the mother may lead to severe illness in pregnancy and may be associated with virus transmission from mother to foetus/newborn.

Since the incidence of this sexually transmitted infection continues to rise and because the greatest incidence of herpes simplex virus infections occur in women of reproductive age, the risk of maternal transmission of the virus to the foetus or neonate has become a major health concern.

On these purposes the Authors of this review looked for the medical literature and pertinent publications to define the status of art regarding the epidemiology, the diagnosis, the therapy and the prevention of HSV in pregnant women and neonate. Special emphasis is placed upon the importance of genital herpes simplex virus infection in pregnancy and on the its prevention to avoid neonatal HSV infections.

## Introduction

Herpes simplex virus (HSV) infection is one of the most common viral sexually transmitted diseases (STD) worldwide [1,2]. Herpes simplex virus type 2 (HSV-2) is the cause of most genital herpes and is almost always sexually transmitted. Herpes simplex virus type 1 (HSV-1) is usually transmitted during childhood via non-sexual contacts. However, HSV-1 has emerged as a principle causative agent of genital herpes in some developed countries [1,3,4]. In the United States (US), HSV-1 is an important cause of genital herpes and its importance is increasing in college students [1,5,6].

The greatest incidence of HSV infections occurs in women of reproductive age, the risk of maternal transmission of the virus to the foetus or neonate has become a major health concern [2,7-11].

Recent findings reveal that first-time infection of the mother is the most important factor for the transmission of genital herpes from mother to foetus/newborn. In fact, the pregnant woman who acquires genital herpes as a primary infection in the latter half of pregnancy, rather than prior to pregnancy, is at greatest risk of transmitting these viruses to her newborn. Additional risk factors for neonatal HSV infection include the use of a foetal-scalp electrode and the age of the mother less than 21 years. Interventions based on these findings led to new management of the pregnant patient with genital herpes prior to pregnancy and to prevention measures to avoid the acquisition of herpes during pregnancy [8].

The Authors of this review looked for the medical literature and pertinent publications to appreciate the importance of genital HSV infection in pregnancy and in neonate. They focused their research on the epidemiology of genital HSV infection, the risks of transmission, the diagnosis, the current therapy and the prevention strategies. For reviewing they used Medline and recent bibliographies.

### Epidemiology of HSV infection, maternal infection and maternal-foetal transmission

HSV-1 and HSV-2 are DNA viruses that belong to *Alphaherpesvirinae*, a subfamily of the *Herpesviridae *family. Both viruses, transmitted across epithelial mucosal cells, as well as through skin interruptions, migrate to nerve tissues, where they persist in a latent state. HSV-1 predominates in orofacial lesions and it is typically found in the trigeminal ganglia, whereas HSV-2 is most commonly found in the lumbosacral ganglia. Nevertheless these viruses can infect both orofacial areas and the genital tract [7].

In recent years, genital herpes has become an increasing common sexually transmitted infection [2,12]. From the late 1970s, HSV-2 seroprevalence in the US has increased by 30%, resulting that one out of five adults is infected [2,13].

Comparing the developing countries, substantially higher rates of HSV2 have been observed in sub-Saharan Africa, where prevalence in adults ranges from 30% to 80% in women and from 10% to 50% in men, finally more than 80% of female commercial sex workers are infected [12]. In South America, available data are mainly for women, in whom HSV2 prevalence ranges from 20% to 40%. Prevalence in the general population of Asian countries shows lower values, from 10% to 30% [3,12].

HSV seroprevalence in patients attending STD clinics varies from 17% in Italy (6% in the general population) to 40% in Australia (14% in pregnant women) [14,15].

Age and sex are important risk factors associated with the acquisition of genital HSV-2 infection. In fact, the prevalence of HSV infection is very low in childhood and early adolescence but it rises with age, reaching the maximum around 40 years [2].

Regarding sex, serological surgery have confirmed that infection is more frequent in women than in men in the general population of US (23,1% in women versus 11,2% in men) and other countries, although in Italy, the seroprevalence is slightly higher in men (6,7%) than in women (4,9%). It is probably due to the younger age of the female group, as well as to the low number of sexual partners for these women, may explain the results [7,16,17]. In fact the strongest association with HSV-2 infection appears related to the number of sexual partners.

The specific geographic distribution can also influence the difference in HSV-2 prevalence [14]. In fact, the seroprevalence found in a STD clinic in Northern Italy is lower than that found among STD clinic attendees in US, Australia and in a previous Italian study, but it is comparable with that found in similar populations within United Kingdom and New Zealand [14]. In addition, ethnicity, poverty, cocaine abuse, earlier onset of sexual activity, sexual behaviour and bacterial vaginosis can facilitate a woman's risk of infection before pregnancy [1,18,19].

Regarding pregnant population, there is a high prevalence of genital herpes. Among Italian pregnant women, the 7.6% seroprevalence observed in Rome is consistent respect to the 8.4% seroprevalence found in Northern Italy in a similar setting [17]. Nevertheless it is lower than that reported among pregnant women in other countries [3,4,20]. For example, in US, approximately 22% of pregnant women are infected with HSV-2, 10% are at risk of acquisition of genital HSV from their infected partners (during periods of asymptomatic viral shedding) and 2% of women acquire genital herpes during pregnancy, placing their newborn at risk for herpes infection [8,10,21]. In Italy, the number of women who acquire HSV infection during pregnancy is about 3% [22]. The acquisition of genital herpes during pregnancy has been associated with spontaneous abortion, intrauterine growth retardation, preterm labour, congenital and neonatal herpes infections [23]. The risk of neonatal infection varies from 30% to 50% for HSV infections that onset in late pregnancy (last trimester), whereas early pregnancy infection carries a risk of about 1% [24]. When primary HSV infection occurs during late pregnancy, there is not adequate time to develop antibodies needed to suppress viral replication before labour. Transmission of HSV from mother to foetus during pregnancy is uncommon; about 85% of perinatal transmission occurs during the intrapartum period [25]. Moreover, studies in HIV-infected pregnant women show that co-infection with HSV increases significantly the risk of perinatal HIV transmission above all in women who had a clinical diagnosis of genital herpes during pregnancy [26].

The newborn could be also infected by HSV-1, that may represent almost one-third of all new genital HSV diagnoses [1]. An increasing proportion of genital herpes infections due to HSV-1 is particularly evident among college-age populations (16–21 years) of the Midwest (US), where it reached about 78% in 2001 (31% a decade earlier) [6]. This result suggested that there is a risk of HSV-1 transmission to newborn when these young women become pregnant and that oral-genital contact is a risk factor for HSV-1 [6]. HSV-1 infection during childhood has declined so that more adolescents and young adults are HSV seronegative when becoming sexually active [8]. This would explain the observed increase in HSV-1 first time infection of the genital tract in this age group.

### Genital herpes: clinical features

Genital HSV infection may be symptomatic or asymptomatic. Symptomatic infection is generally described as genital herpes and include primary, first-episode and recurrent herpes outbreaks. Primary genital herpes is usually the most serious event for the individual, especially in pregnancy, since it can cause the most severe neonatal disease. Moreover, it is defined as first-episode of genital herpes where the patient has no antibody against HSV-1 and HSV-2 [2].

Primary symptomatic genital herpes, that occurs after an incubation of a period of 2–20 days, is usually important and prolonged (up to 21 days) [2,11]. Within women it causes blistering and ulceration of the external genitalia and cervix leading to vulval pain, dysuria, vaginal discharge and local lymphadenopathy [9]. Vesicular and ulcerative lesions of the internal thigh, buttocks, perineum or in perianal skin are also been observed. In men the lesions typically develop on the glans, but also on the penis, internal thigh, buttocks or in perianal skin. Both in man and in woman primary infection may be complicated by systemic symptoms such as fever, headache and myalgia (38% in men, 68% in women) and occasionally meningitis and by autonomic neuropathy resulting in urinary retention, mainly in women [9,11]. Meningitis has been found in 42% of primary HSV-2, 12% of primary HSV-1 infections and 1% of recurrent infections [11]. Nevertheless, pre-existing HSV-1 antibodies can alleviate clinical manifestations of subsequently acquired HSV-2 [1]. In some cases, systemic clinical findings may be the only presenting symptoms of infection and in more than half of patients, primary infection goes unnoticed [9].

The most important HSV infection during pregnancy is the primary genital HSV infection, although, in the majority of pregnant women, the first manifestation of genital herpes is not a primary infection [9].

Primary HSV infections in pregnant women can result in more severe diseases than that in non-pregnant ones. In particular, gingivostomatitis and vulvovaginitis herpetica tend towards dissemination. As a result, women can develop disseminated skin lesions associated with visceral involvement such as hepatitis, encephalitis, thrombocytopenia, leucopoenia and coagulopathy [9]. Although disseminated HSV infection is uncommon in pregnancy, the mortality is about 50%. In particular, pregnant women with primary mucous membrane infection during the third trimester, have an increased risk for dissemination and they could transmit HSV to their babies during vaginal delivery [9].

Recurrent episodes of HSV infection are characterized by the presence of antibody against the same HSV type and the herpes outbreaks are usually mild (7–10 days) with less severe symptoms than the first episode. Prodromal symptoms (itching, tingling, neuralgia) may occur hours or days before a recurrent herpes episode [2,27]. The great majority of recurrent genital herpes is due to HSV-2 because this virus reactivates more frequently than HSV-1 [2,7,9].

The apparently asymptomatic phases between clinical outbreaks of genital herpes are important, since HSV can reactivate periodically in latently infected cells of sensory ganglia travelling via the neuronal axons back to the genital mucosa, without clinical signs or symptoms. This mechanism is known as asymptomatic virus shedding [2,11]. The majority of sexual HSV transmission occurs during asymptomatic periods because the patients are unaware of asymptomatic virus shedding [28]. Moreover, asymptomatic shedding has been shown to be higher in women with HSV-2 infection compared with those with HSV-1 (7% versus 2% respectively) [2].

Although there is a small risk of vertical transmission, recurrent genital herpes must be regarded as the most common cause of neonatal infections and the passage through an infected birth canal is the most probable route of transmission [9]. In recurrent infections associated with clinical symptoms, the risk of neonatal disease is reduced dramatically by caesarean section [10,29]. Transmission of HSV by women with asymptomatic viral shedding is of greater significance, since neonates mostly acquire infection without being recognized [9].

### Management of pregnant women with a first or recurrent episode of genital herpes

#### Diagnostic procedures

Diagnosis of genital HSV infections is often complicated because non-classical presentations are common or clinical signs are mild and non-specific. Moreover, HSV infection is characterized by clinical outbreaks followed by asymptomatic periods within HSV transmission is possible. Therefore, it is necessary to improve the recognition and hence diagnosis of genital herpes, because a correct laboratory diagnosis is important for clinical management, counselling, treatment, management of pregnancy and assessment of the risk of transmission [2,11].

The HSV infection may be identified directly by detection of the virus or one of its components (Table 1), or indirectly by assaying for specific serum antibodies of the viruses (Table 2) [2,30-37].

**Table 1:** Direct methods for HSV diagnosis

**Method**	**Tissue sampled**	**Sensitivity**	**Specificity**	**Advantages**	**Disadvantages**
Virus isolation by cell culture^1^	Skin/mucosal lesions (stage):				Specialized laboratories
	- vesicular content	>90%		Gold standard	Virus transport medium
	- ulcers	95%	~100%	Simplicity of sampling	Transport rapid, cooled, protected from light
	- scabs	70%		Virus typing	Results in 2/7 days
	- mucosa without lesions	30%		Resistance phenotype determination	Not suitable for CFS
		Unknown			Arrangement with laboratory necessary
	Biopsies				
	Conjunctival smear/corneal				
	Neonates				

Cytologic diagnosis (Tzanck's smear)^35^	Skin/mucosal lesions	73–100%	100%	Easy, quick, reproducible and inexpensive	Optimal lesions are fresh, intact bisters of 1/3 days' duration
	Biopsies				
	Conjunctival smear/corneal				

IF (detection of infected cells)^30^	Smears, tissue sections, smears from base of vesicle	41–70%	>95%	Rapid (<4 h possible) Typing possible	Fresh vesicles
					Specialised laboratories
					Technically demanding
					Not standardized

Virus antigen detection by EIA o ELISA^30^	Smears from lesions, vesicular content with base of vesicle	41–80%	80%	Simplicity of sampling	Suitable only for fresh vesicles
				Does not require the integrity of the specimen	
				Rapid (<4 h possible)	
				Typing possible	

				**PCR**:	
				Most sensitive method	
Virus DNA detection by **PCR**^30 ^or **Real-time PCR**^31^	CSF	9798%	~100%	Result within 24–48 h	Only in specialised laboratories
	Aqueous or vitreous humour			Virus typing and resistance genotyping	Not standardised
				Method of choice for CSF	Not validated for all samples
					Risk of contamination (PCR)
				**Real-time PCR**:	High costs (real-time PCR)
	Skin lesions, vesicular content or mucosa without lesions			Rapid amplification	
				Quantitative analysis	
				Reduced risk of contamination	
				Method of choice for skin lesions	

**Table 2:** Indirect methods for HSV diagnosis

**Method**	**Tissue sampled**	**Sensitivity**	**Specificity**	**Advantages**	**Disadvantages**
				Distinguish between HSV-1 and 2	
Western Blot^2^	Serum	~100%	~100%	Detect early seroconversion to HSV-2 in patient with prior HSV-1 infection.	Not commercially available Expensive 2–3 days for results

				Commercially available	
EIA^2^	Serum	93–98%	93–98%	Distinguish between HSV-1 and HSV-2	Lack of sensitivity (compared to amplified tests)^2^

	Serum			Less expensive than western blot^2^	Commercially available only for HSV-2^2^
Point of care tests^2^	Capillary blood^37^	96%	87–98%	Accurate results rapidly (6 min.)^37^	Expensive^36^
				Easily performer^37^	Not for large volume screening^36^
				Detects seroconversion within 4 weeks of presentation of 80% of patients with HSV-2 episodes^37^	Complexity nonwaived (moderate)^36^

Direct site-specific methods, such as virus or antigen detection, are the most relevant in patients with active, vesicular lesions at or near a genital site. When lesions have scabbed or are not evident, HSV-1 or HSV-2 infection can be diagnosed indirectly by detection of type-specific IgG against the glycoprotein G of HSV-1 (gG-1) or the glycoprotein G of HSV-2 (gG-2) [2,30]. Indirect (serological) testing can provide useful information in symptomatic patients when direct methods have yielded negative results. Although serological testing cannot reveal the onset of HSV infection or identify the locus of shedding [7], it allows identification of HSV infection when direct virus detection methods are not viable or when evidence of seroconversion is required [2]. Moreover, indirect approaches are useful to determine the type of recurrence. In general, genital HSV-1 causes a severe initial outbreak but fewer recurrences than HSV-2 [7]. However, type-specific testing is useful but not essential, because treatment regimens do not vary by virus type [7].

#### Therapeutic measures

Pregnant women with a first clinical episode or a recurrence may be treated with acyclovir or valacyclovir at the recommended dosages (Table 3). Since acyclovir and valacyclovir are not officially approved for treatment of pregnant women, patients should be informed to give consent before the administration [9]. However, no increase of foetal abnormalities was ascribed to these treatments, although long-term outcomes were not evaluated [38-40].

**Table 3:** Antiviral treatment of genital herpes in pregnancy

	**First episode**			**Recurrent episodes**		
	
**Pregnancy**	***Antiviral drug***	***Recommended daily dosage***	***Length of therapy***	***Antiviral drug***	***Recommended daily dosage***	***Length of therapy***
**Episodic treatment**	Acyclovir	Orally: 5 × 200 mg	10 days	Acyclovir	Orally: 5 × 200 mg	5 days
	Valacyclovir	Orally: 2 × 500 mg	10 days	Valacyclovir	Orally: 2 × 500 mg	5 days

**Suppressive treatment**	Acyclovir	Orally: 3 × 400 mg		Acyclovir	Orally: 3 × 400 mg	
	Valacyclovir	Orally: 2 × 250 mg	From week 36 until delivery	Valacyclovir	Orally: 2 × 250 mg	From week 36 until delivery

Randomised studies have shown that suppressive treatments with acyclovir and valacyclovir from 36th week of pregnancy until delivery, significantly reduces the frequency of clinical manifestations and the virus shedding at the time of delivery decreasing the need for caesarean delivery and probably the risk of vertical transmission (Table 3) [41-45].

#### Mode of delivery

**Figure 1:**
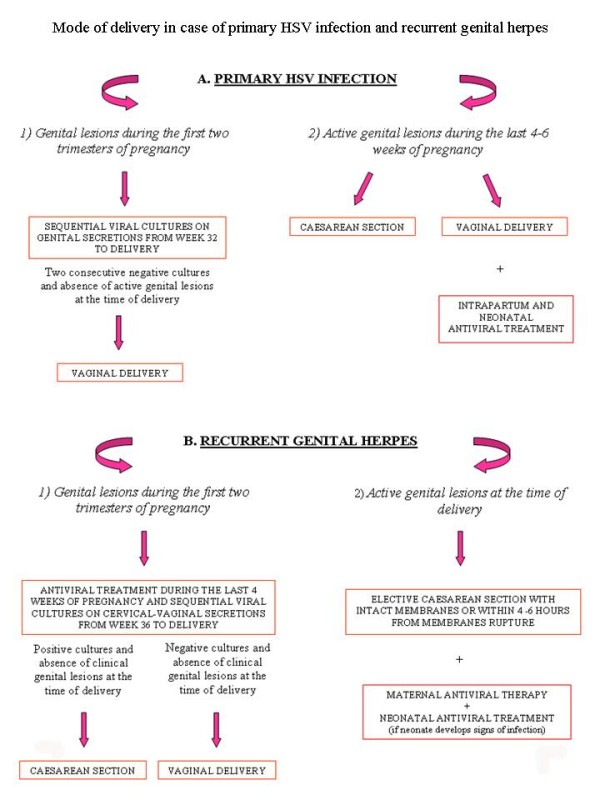
**The figure resumes in a schematic diagram the mode of delivery in HSV primary infection (A) and in recurrent genital herpes infections (B)**.

When primary infection is acquired during the first two trimesters of pregnancy, it is advisable to carry out sequential viral cultures on genital secretions from 32th week of gestation [22]. If two consecutive cultures result negative and there are no active herpetic genital lesions at the time of delivery, it is possible to perform a vaginal delivery (Fig. 1, section A1). If seroconversion is completed at the time of delivery, caesarean section is not required since the risk of HSV transmission to the foetus is low and the neonate should be protected by maternal antibodies [9,22].

If primary genital infection is acquired during the third trimester of pregnancy, the optimal way of proceeding is not well defined. Most guidelines propose caesarean section for women developing a primary clinical infection within the last 4–6 weeks of gestation, because they can not complete their seroconversion prior to the time of delivery and therefore they could infect the neonates [9,23,30,46-48]. When vaginal delivery is irreversible, since the risk of vertical transmission is high (41%), a maternal and neonatal intravenous acyclovir therapy is recommended (Fig. 1, section A2) [22].

For women who present an episode of recurrent genital herpes several weeks before the expected delivery date, a suppressive therapy with acyclovir or valacyclovir is recommended during the last 4 weeks of pregnancy and viral cultures on cervical-vaginal secretions from 36th week of gestation are required [22,47]. Furthermore, when there are no clinical herpes lesions but virus detection tests result positive at the time of delivery, an elective caesarean section is indicated [10,30]. On the contrary, if all viral cultures are negative and there are no genital herpetic lesions at the time of delivery, it is possible to perform a vaginal delivery (Fig. 1, section B1) [47].

Finally, since active genital HSV lesions are present or prodromal symptoms occur at the onset of delivery and consequently the risk of viral exposure to the infant is high, a caesarean section should be performed as quickly as possible within 4–6 hours after membranes rupture if foetal lungs are mature [22,30,49]. When foetal lungs are immature, there are no established guidelines [9,30].

A cesarean delivery before ruptured membranes virtually eliminates the risk of intrapartum transmission to the infant [7,10], although it does not completely remove the risk of HSV transmission [10,50]. An antiviral treatment with acyclovir is recommended to the mother and eventually to the newborn (Fig. 1, section B2) [30].

### Neonatal hsv infections

#### Mode of acquisition and clinical manifestations

HSV infection of the newborn can be acquired in utero, intrapartum and postnatally. The mother is the most common source of infection for the first two routes of viral transmission [51].

Intrauterine HSV infection is a rare disorder and accounts for 5% of HSV infections in neonates. The highest risk of intrauterine infection has been observed in pregnants (about 50%) who develop disseminated HSV infections and 90% of those are related to HSV-2. Both primary and recurrent maternal infection can result in congenital disease, even if the risk after recurrent infection is small. Intrauterine viral transmission is highest during the first 20 weeks of gestation leading to abortion, stillbirth and congenital anomalies in infants who survive [9]. The perinatal mortality is 50% [11].

In 85–90% of neonatal HSV infections, HSV is acquired at the time of delivery and 5–10% are caused by early postnatal viral acquisition. 70–85% of neonatal HSV infections are caused by HSV-2, whereas the remaining cases are due to HSV-1 [50]. Usually, an infection with HSV-2 carries a graver prognosis than that caused by HSV-1 [7,52]. The estimate rate of occurrence ranges widely from 1/3200 to 1/20000 of life births [10,53-56].

The disease transmission to the newborn is dependent on the type of maternal genital infection at the time of delivery. In fact, neonatal herpes is much more frequent (50%) in babies from mothers with a primary HSV infection respect to babies from mothers with recurrent HSV infection (<3%) [22,57]. However, most neonatal HSV infections (about 70%) result from exposure to asymptomatic genital HSV infection in the mother near delivery [43].

The prolonged rupture of membranes is a risk marker for acquisition of neonatal infection [51]. Women with active genital lesion at the time of labor usually have their infants delivered by caesarean section. Nevertheless, it is not clear whether this procedure reduces HSV transmission to the newborn [10]. Finally, invasive obstetric procedures and the use of foetal scalp monitors appear to have a great effect on neonatal herpes transmission because they can create a site of inoculation of the virus [54,58-62].

The clinical presentation of infants with neonatal HSV infection, that is almost invariably symptomatic and frequently lethal, is a direct reflection of the site and extent of viral replication [51]. Congenital intrauterine infection, that usually is identified within the first 48 hours following birth, is characterized by skin vesicles or scarring, eye lesions (chorioretinitis, microphthalmia, cataract), neurologic damage (intracranial calcifications, microcephaly, seizures, encephalomacia), growth retardation and psychomotor development [9]. Infants infected intrapartum or postnatally by HSV can be divided into three major categories: 1) HSV disease localized to the skin, eye, and/or mouth; this syndrome is associated with a low mortality but it has a significant morbidity and it may progress to encephalitis or disseminated disease if left untreated [58]; 2) HSV encephalitis with or without skin, eye, and/or mouth involvement which causes neurologic morbidity among the majority of survivors [63]; 3) disseminated HSV which manifests as severe multi-organ dysfunction (including central nervous system, liver, lung, brain, adrenals, skin, eye and/or mouth) and has a mortality risk that exceeds 80% in absence of therapy [51,58].

At diagnosis, symptoms are found with the following frequency: skin vesicles 68%, fever 39%, lethargy 38%, seizures 27%, conjunctivitis 19%, pneumonia 13%, disseminated intravascular coagulation 11%. Symptoms may occasionally be present at birth, but occur in 60% later than 5 days after birth and sometimes are present after 4–6 weeks of life [30,63].

Localized infections have been found in 50% of the affected neonates, involvement of the central nervous system (CNS) in 33% and disseminated infections in 17% of the cases [9,30]. Several studies have demonstrated that disseminated HSV infections are characterized mainly by liver and adrenals failure associated with shock symptoms and disseminated intravascular coagulopathy [51,52,64]. Other symptoms of HSV disseminated infection include irritability, seizures, respiratory distress, jaundice and frequently the characteristic vesicular exanthem that is often considered pathognomonic for infection. However, over 20% of infants with disseminated infection do not develop skin vesicles during the course of their illness. Encephalitis appears to be a common component of this infection form, occurring in about 60–75% of infants with disseminated HSV infection. Mortality in the absence of therapy exceeds 80% [51].

Despite the availability of antiviral drugs for treatment of neonatal HSV infections, the outcome remains poor, particularly for babies with disseminated multi-organ infections or manifestations of CNS [55]. Infection of the CNS, alone or in combination with disseminated disease, is characterized by neonatal hemorrhagic-necrotizing encephalitis that manifests as lethargy, seizures (both focal and generalized), irritability, tremors, poor feeding, temperature instability, bulding fontanelle and pyramidal tract signs [9,51]. Although the mortality rate is only 5% for neonates with encephalitis, over 50% of survivors are left with significant neurological impairment, whereas for children with disseminated multi-organ disease, the mortality rate approaches 30% and nearly 20% of survivors have neurological impairment [55]. After a neonatal herpes infection, cutaneous recurrences may occur [65]. Moreover, the outcome is correlated with the virus type and disease classification. In particular, for treated babies with skin, eye and mouth involvement attributed to HSV-1, there are no consequences, whereas 3% of those with skin disease caused by HSV-2 subsequently develop neurological complications. Regarding infants with encephalitis, the neurological outcome is significantly better for HSV-1 respect to HSV-2 infection. In fact 25% of babies with HSV-1 infection show severe impairment, compared with 55% with HSV-2 infection. The outcome is reversed for babies with disseminated disease. In this circumstance, 70% of babies with HSV-1 infection die or have severe neurological impairment compared with 50% of babies infected by HSV-2 [55].

#### Diagnostic procedures

When perinatal HSV exposure is known, it is advisable to collect and to analyze swabs from neonate's conjunctiva, oropharynx and rectum within 24–48 hours after delivery. Moreover, these neonates must be monitored closely up to 4–6 weeks of age. If the neonate exhibits suspicious symptoms of infection, cultures of vesicular, conjunctival, oropharyngeal, stool/rectal swabs, urine and blood must be performed. In addition, HSV-PCR analysis on cerebrospinal fluid (CSF) and routine laboratory tests should be carried out (Table 1). Cerebral imaging and/or ophthalmological examination should be performed [9,30,50].

#### Antiviral therapy and prognosis

All infants with a suspected or diagnosed HSV infection must be treated with an intravenous therapy with acyclovir (60 mg/kg/day). The starting time of treatment is crucial for prognosis, especially in case of disseminated infections. HSV infections localized to skin, eyes and mucous membranes are treated for 14 days, whereas CNS or disseminated infections required 21 days of therapy (Table 4) [9,30,50].

**Table 4:** Antiviral treatment of neonatal HSV infection

**Infants**	***Antiviral drug***	***Recommended daily dosage***	***Length of therapy***
			Localised infections: 14 days
Treatment of neonatal hsv infection	Acyclovir	Intravenously: 3 × 10–20 mg/kg	CNS or disseminated infections: 21 days

Suppressive treatment of cutaneous recurrences after neonatal herpes	Acyclovir	Orally: 2–3 × 300 mg/m^2^	For weeks to months

Source: Swiss Herpes Management Forum, 2004

Suppressive antiviral treatment with acyclovir is indicated when cutaneous recurrences are observed after neonatal HSV infection (Table 4) [9,30,66]. In case of ophthalmic herpes, infection monitoring should be carried out in order to rule out keratitis [30].

Although high-dose of intravenous acyclovir for a sufficient period has been proven to be effective [30,67], neonatal HSV infection is still associated with high residual lethality and morbidity because acyclovir administration may suppress but not eradicate the virus in exposed infants [50].

Localised form heals without sequelae whereas the CNS form is lethal in 6% of cases leaving 69% of permanent late sequelae. The disseminated infection takes a lethal course in 31% and has late sequelae in 17% of cases [30,67].

### Prevention of neonatal hsv infections

The high rate of undiagnosed or asymptomatic HSV infections complicate the prevention [7]. In order to avoid the majority of neonatal herpes cases, identification of the *at-risk mother *is the goal. The first and most important step is the determination of the pregnant women serostatus to establish their susceptibility to the infection during early pregnancy [8]. However, current recommendations of the American College of Obstetricians and Gynecologists (ACOG) do not include universal testing because at the present time, type-specific serologic tests are not widely available and their reliability is questionable [8,50]. The most effective measure to prevent perinatal herpes infections is to avoid viral exposure to the neonate when primary genital herpes develops in late pregnancy whereas the risk of severe neonatal infection is small in recurrent episodes [9]. A history of HSV infection in all pregnant women and their partner should be obtained at the first prenatal visit [47,50,68]. Women with a negative personal history of HSV and especially those with a positive history in the male partner, should be strongly advised to have no oral and sexual intercourse at the time of recurrence in order to avoid infection (in particular during the third trimester of gestation) [9,50]. Moreover, use of condoms throughout pregnancy should be recommended to minimize the risk of viral acquisition, although the male partner has no active lesions [9,50]. However, condoms are not a complete barrier for the genital region [7]. Prophylactic administration of acyclovir or valacyclovir in the third trimester of pregnancy should be provided to all pregnants with frequent genital herpes outbreaks and with active genital HSV infection near term or at the time of delivery [7,8,41-43,50,69]. A careful examination of the vulva, vagina and cervix should be performed on any woman who presents signs or symptoms of HSV infection at the onset of labour. Artificial rupture of membranes should be avoided [8,50]. All pregnants who have a suspected active genital HSV infection or prodromal symptoms of HSV infection should undergo caesarean section, although membranes are intact [50]. On the contrary, when genital herpes lesions are not present, caesarean delivery is not required but lesions should be covered with an occlusive dressing before vaginal delivery [47,50]. It is important to remember that foetal scalp electrodes monitoring during labour and vacuum or forceps delivery should be used only if necessary, since these practices appear to increase the risk of HSV transmission [8,50].

Neonates, born to women with active genital lesions, with a confirmed or suspected HSV infection should be isolated, managed with contact precautions to avoid direct contact with skin and mucosal lesions, excretions, body fluids and immediately treated with intravenous acyclovir [9,50]. Since neonatal herpes can also be acquired postnatally, postpartum women, family members and nursery personnel with active herpetic lesions of the mouth, skin or breast should take necessary precautionary measures to prevent direct contact with the neonate and/or should be excluded from the neonatal unit until the lesions are fully healed [9].

### HSV vaccine studies

The development of vaccines against herpesviruses has major public health importance in both immunocompetent and immunocompromised populations. Because these viruses establish latent infections capable of subsequent reactivation, both immunotherapeutic and prophylactic vaccine strategies are needed.

About prophylactic vaccines, partially effective prophylactic vaccines may still be useful if they shift the threshold of infection, or if they prevent or improve disease. They could reduce HSV2 incidence by preventing infection or by reducing the shedding or clinical recurrences in a HSV2-infected individual. On the other hand, these vaccines could increase HSV2 incidence reducing symptomatic signs of disease without effect on viral shedding. In particular, the Chiron-gD2gB2-MF59 vaccine provided only temporary protection lasting a few months, whereas the GlaxoSmithKline (GSK)-gD2-alum-MPL prophylactic vaccine had no effect in men or HSV1 positive women although in HSV1 seronegative women the risk of HSV2 infection and disease was reduced. A further trial of the GSK vaccine in HSV1 negative women is ongoing [70].

Numerous approaches including subunit vaccines, peptide vaccines, live virus vectors and DNA vaccine technology have been used in developing both prophylactic and therapeutic vaccines, since several antiviral therapies are available to control disease and spread, but these are not completely effective and do not affect latent virus [71].

A range of vaccine formulations has been devised, largely as a result of the rapid growth in knowledge in molecular microbiology and genetic engineering, including live and inactivated whole virus vaccines and subunit vaccines consisting of recombinant viral glycoproteins in various adjuvants [72].

Although animal studies on vaccination strategies to prevent genital and neonatal herpes may be promising, clinical trials of HSV-2 vaccines in humans have failed to prove efficacy. In a previous study, an HSV-2 glycoprotein D vaccine using alummorpholine (MPL) as adjuvant, induced protection from clinical disease (73%) and overall HSV-2 transmission (about 40%) [73]. Nevertheless, the protective effect of the MPL vaccine was seen only in women who were HSV-1 and HSV-2 seronegative and there was no protection among men or among HSV-1 seropositive women [3].

In conclusion, many prophylactic and therapeutic vaccination approaches have been explored but no effective vaccine is presently available.

## Conclusion

A large body of information on the transmission of herpes from male to pregnant partner, on the mode of transmission from mother to newborn, mainly by maternal first-time infection in the third trimester of pregnancy, have been published in literature.

Since the increasing prevalence of genital HSV infection and apparent increase in the incidence of neonatal herpes, we have focused our attention on prevention of maternal-foetal transmission as well as on the management of infected pregnant women and neonate. Further studies are needed to monitor the changing HSV-1 and HSV-2 trends and to develop effective strategies to prevent HSV infection. Finally, the major vaccine strategies under development should take in an account the three important features of herpesviruses: the viral latency, the herpes immune escape and the high seroprevalence.

## Competing interests

The authors declare that they have no competing interests.

## Authors' contributions

EA, DF, MM, AB, VB, FC and VP conceived of the study, and participated in its design and coordination. All authors read and approved the final manuscript.
